# Cryo-EM Structures of CusA Reveal a Mechanism of Metal-Ion Export

**DOI:** 10.1128/mBio.00452-21

**Published:** 2021-04-05

**Authors:** Mitchell A. Moseng, Meinan Lyu, Tanadet Pipatpolkai, Przemyslaw Glaza, Corey C. Emerson, Phoebe L. Stewart, Phillip J. Stansfeld, Edward W. Yu

**Affiliations:** a Department of Pharmacology, Case Western Reserve University School of Medicine, Cleveland, Ohio, USA; b Department of Biochemistry, University of Oxford, Oxford, United Kingdom; c School of Life Sciences & Department of Chemistry, University of Warwick, Coventry, United Kingdom; Harvard Medical School

**Keywords:** CusA, antimicrobial resistance, cryo-EM, efflux pump, resistance-nodulation-cell division

## Abstract

Gram-negative bacteria utilize the resistance-nodulation-cell division (RND) superfamily of efflux pumps to expel a variety of toxic compounds from the cell. The Escherichia coli CusA membrane protein, which recognizes and extrudes biocidal Cu(I) and Ag(I) ions, belongs to the heavy-metal efflux (HME) subfamily of RND efflux pumps. We here report four structures of the trimeric CusA heavy-metal efflux pump in the presence of Cu(I) using single-particle cryo-electron microscopy (cryo-EM). We discover that different CusA protomers within the trimer are able to bind Cu(I) ions simultaneously. Our structural data combined with molecular dynamics (MD) simulations allow us to propose a mechanism for ion transport where each CusA protomer functions independently within the trimer.

## Abstract (precis)

The bacterial RND superfamily of efflux pumps mediate resistance to a variety of biocides, including Cu(I) and Ag(I) ions. Here we report four cryo-EM structures of the trimeric CusA pump in the presence of Cu(I). Combined with MD simulations, our data indicate that each CusA protomer within the trimer recognizes and extrudes Cu(I) independently.

## INTRODUCTION

In Gram-negative bacteria, efflux systems of the resistance-nodulation-cell division (RND) superfamily significantly affect both the intrinsic and acquired tolerance levels of the organism to antimicrobial agents and toxic metal ions, including Cu(I) and Ag(I) (1). Typically, an RND efflux pump works in conjunction with a periplasmic membrane fusion protein and an outer membrane channel to form a functional tripartite protein complex. Escherichia coli harbors seven of these efflux pumps that can be characterized into two distinct classes, the hydrophobe-amphiphile efflux RND (HAE-RND) and the heavy-metal efflux RND (HME-RND) families (1).

The HAE-RND efflux pumps have been studied extensively. Several X-ray and cryo-electron microscopy (cryo-EM) structures have been determined within this family of membrane proteins, including E. coli AcrB (2–6), Pseudomonas aeruginosa MexB (7), Neisseria gonorrhoeae MtrD (8, 9), Campylobacter jejuni CmeB (10), and Acinetobacter baumannii AdeB (11). It has been proposed that these RND efflux pumps utilize a rotating mechanism, where the three subunits within the trimeric pump are synchronized and coordinated to advance the transport cycle for drug extrusion (3, 6, 12). However, a direct observation of transport dynamics using single-molecule fluorescence resonance energy transfer (FRET) imaging indicated that each protomer of the trimeric CmeB multidrug efflux pump undergoes uncoordinated conformational transitions and function independently of each other (10). Therefore, the action mechanism of these HAE-RND multidrug efflux pumps still remains elusive.

In contrast, the structural and molecular mechanisms of the HME-RND efflux pumps are far less studied. At present, only two crystal structures belonging to the HME-RND pumps are available. These pumps are the E. coli CusA (13) and Cupriavidus metallidurans CH34 ZneA (14) heavy-metal efflux transporters. In addition, the co-crystal structure of the CusA-CusB transporter-adaptor complex has been reported (15), providing the first structural evidence that a trimeric efflux pump within the RND superfamily interacts with six adaptor molecules to assemble and function.

To elucidate the mechanisms of heavy-metal recognition and extrusion of the E. coli CusA efflux transporter, we define cryo-EM structures of this membrane protein embedded in lipidic nanodiscs in the presence of Cu(I) ions. Cryo-EM is an imaging technique that snapshots single-particle images at random orientations in a frozen-hydrated state. It has a capability of recording different conformational states of proteins and other biomacromolecules within a single sample (16, 17). Recently, it has also been shown that this technique is capable of simultaneously solving structures of a variety of membrane proteins from a heterogeneous, impure sample (18). With the cryo-EM approach, we are able to observe detailed structural information of various transient states that the CusA pump may need to adopt in order to recognize and extrude metal ions. We here present four cryo-EM structures of the trimeric CusA efflux pump, either alone or bound with Cu(I). We observe that CusA can form both symmetric and asymmetric trimers, with different CusA protomers within the trimer able to bind Cu(I) simultaneously. We also conduct molecular dynamics (MD) simulations to demonstrate transitions between different states captured from the cryo-EM structure and observe a proton permeation pathway at the transmembrane domain. On the basis of our findings, we propose a mechanistic model of transport that suggests each CusA protomer functions independently within the trimer.

## RESULTS

### Structural determination of the CusA heavy-metal efflux pump.

Previously, the structures of CusA, both in the absence and presence of Cu(I) or Ag(I), have been determined by X-ray crystallography (13). In the absence of Cu(I) or Ag(I), the structure of apo-CusA presents the “resting” state conformation, where the periplasmic cleft is closed. However, upon the addition of metal ion, both the CusA-Cu(I) and CusA-Ag(I) structures are characterized by the open cleft conformation, where the PC2 subdomain is found to swing away from the PC1 subdomain by 30^°^ when compared with the apo-CusA structure. This conformational shift allows the periplasmic cleft to open (13). In addition, a single Cu(I) or Ag(I) is found to bind in the middle of the transient methionine triad M573-M623-M672 situated deep inside the cleft. The CusA-Cu(I) and CusA-Ag(I) structures are nearly identical to each other and represent the “binding” state of the CusA pump.

To continue to elucidate the molecular mechanism of the HME-RND efflux pumps, we decided to solve cryo-EM structures of CusA embedded in nanodiscs in the presence of Cu(I). As the approach of cryo-EM captures single-particle images in a frozen solution state, these images should reflect the different conformations that the CusA pump can possibly achieve in the free solution environment before being frozen. These cryo-EM images should contain critical structural information of different transient states that the CusA pump must go through during the transport cycle. Extensive classification of the single-particle cryo-EM images of the CusA-Cu(I) complex revealed that there were four distinct populations of the CusA particles with different conformations that coexist in the nanodisc sample. Surprisingly, two of these structures illustrated that the trimeric CusA pump assembles as symmetric trimers, where either the three CusA protomers within the trimer are bound by Cu(I), or none of these CusA protomers are occupied by metal ions. The other two structures represent asymmetric trimers, where either one or two CusA protomers within the trimer are bound by Cu(I).

### Structure of trimeric CusA with two closed periplasmic clefts and one open periplasmic cleft.

**FIG 1:**
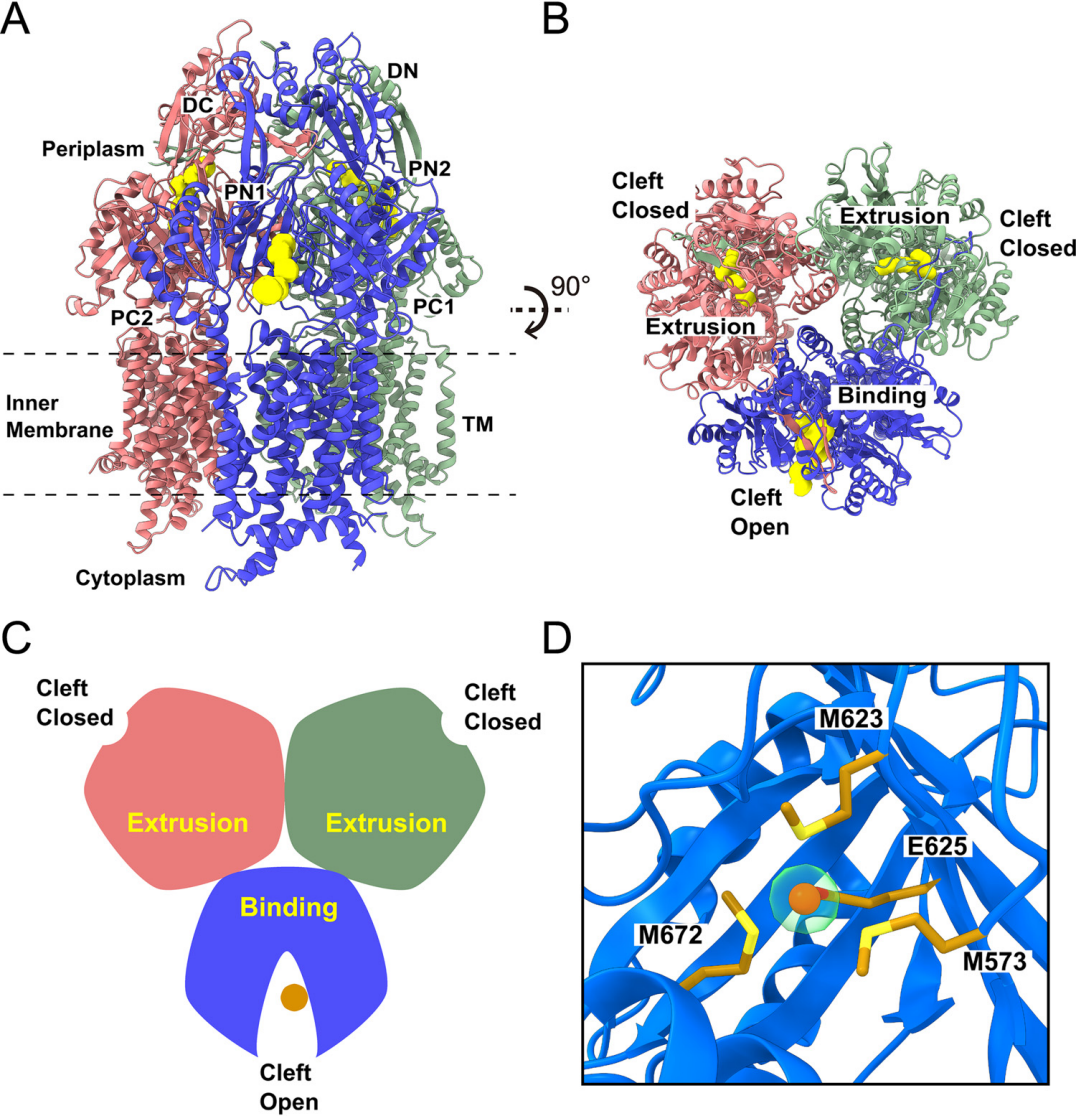
Cryo-EM structure of the CusA trimer in the EEB state. (A) Ribbon diagram of the CusA trimer with two closed cleft “extrusion” state protomers and one open cleft “binding” state protomer viewed from the membrane plane with the “extrusion” and “binding” channels shown in yellow. (B) Ribbon diagram of the EEB CusA trimer viewed from the top of the periplasmic domain illustrating the channels (colored yellow) formed in the “binding” and “extrusion” states of the CusA protomers. (C) A cartoon displaying the conformation of the CusA trimer in the EEB form viewed from the top of the periplasmic domain. In panels A, B, and C, the two “extrusion” protomers and one “binding” protomer of CusA are colored pink, green, and blue, respectively. The bound Cu(I) ion in the “binding” protomer is represented as a dark orange circle. (D) The binding site of the Cu(I) ion (dark orange sphere) within the open periplasmic cleft of the “binding” protomer (blue). The bound Cu(I) ion is coordinated by residues M573, M623, E625, and M672 (gold sticks). The density of bound Cu(I) is shown in transparent light green.

The most abundant conformation of trimeric CusA in our cryo-EM sample represents the transient state with two of the periplasmic clefts formed by subdomains PC1 and PC2 closed and the third cleft completely open. We collected a total of 75,703 single-particle projections for this class of images and determined the structure to a resolution of 2.82 Å (Fig. 1; see also Fig. S1 and Table S1 in the supplemental material). The structure of this conformational state of CusA has two protomers with their periplasmic clefts closed and appear identical. These two protomers represent the “extrusion” form of CusA, as a channel for extrusion is found in each protomer, similar to those found in the HAE-RND efflux pumps AcrB (3, 6), CmeB (10), and MtrD (9) (Fig. 1A to C and Fig. S1). The conformations of these two CusA protomers are also quite distinct from the structures of the three identical protomers of apo-CusA, as determined by X-ray crystallography in the absence of Cu(I). This apo form corresponds to the “resting” state of the CusA pump. Superimposition of an “extrusion” protomer from the cryo-EM structure of CusA onto a “resting” protomer from the crystal structure of apo-CusA results in a root mean square deviation (r.m.s.d.) of 1.5 Å (for 1,029 Cα atoms). For the third CusA protomer, an elongated channel is observed within its periplasmic domain, where this channel leads through the opening of the periplasmic cleft and allows the interior of the protomer exposure to solvent (Fig. 1A and B). This protomer depicts the “binding” state of the membrane protein, where its conformation is comparable to those “binding” protomers identified in AcrB (3, 6), CmeB (10), and MtrD (9). The cryo-EM structure of this “binding” protomer is also nearly identical to the conformation of those protomers obtained from the X-ray structures of the CusA-Cu(I) and CusA-Ag(I) complexes (13). Superimposition of this “binding” protomer to a protomer of the X-ray structure of CusA-Cu(I) gives rise to an r.m.s.d. of 0.8 Å (for 1,029 Cα atoms). It should be noted that the conformations of the transmembrane domain of the CusA-Cu(I) complex in the cryo-EM and X-ray structures are nearly identical, suggesting that the nanodiscs and detergent micelles do not significantly alter the conformation of the transmembrane helices. The assignments of the CusA protomers are shown in Fig. S2.

10.1128/mBio.00452-21.1 FIG S1 Cryo-EM analysis of CusA. (A) Processing of 6,772 micrographs to obtain initial pool of 1,769,806 particles and 2D classification selecting for 549,454 particles. (B) 3D classification results. (C) Reconstruction of trimeric CusA gave rise to four different structures (EEE, EEB, EBB, and BBB). The PDB IDs for the structures of EEB, EBB, EEE, and BBB are 7KF7, 7KF8, 7KF5, and 7KF6, respectively. Each panel contains the cryo-EM analysis of the indicated CusA structure with a side view of the cryo-EM map density for the CusA state composed of the individual protomers (pink, green, and blue) assembled as a trimer into a lipid nanodisc (gray). Following is the cryo-EM map of the CusA trimer viewed from the top of the periplasmic domain beside representative 2D classes and the gold-standard Fourier shell correction (GS-FSC) resolution of the cryo-EM map. Download FIG S1, JPG file, 2.6 MB. Copyright © 2021 Moseng et al. 2021 Moseng et al. https://creativecommons.org/licenses/by/4.0/ This content is distributed under the terms of the Creative Commons Attribution 4.0 International license.

10.1128/mBio.00452-21.2 FIG S2 CusA protomer state classification. Different states of the CusA protomers are classified by (A) the conformation of the periplasmic cleft as measured by the distance between residues L658 (at the right side of the cleft) and L714 (at the left side of the cleft), (B) the size of the exit site of the extrusion channel as measured by the distance between residues P54 and A754, which form this exit site, and (C) the conformation of the proton relay residues, including D405, E938, and K984, in the transmembrane domain. In panel A, the distance between L658 and L714 is 20.4 Å for the “binding” protomer (with the periplasmic cleft open) of the EEB structure (PDB ID 7KF7). This distance is 9.9 Å for the “extrusion” protomer (with the periplasmic cleft closed) of the EEE structure (PDB ID 7KF5). The L658-L714 distance is 9.9 Å for the “resting” protomer (with the periplasmic cleft closed) of the X-ray structure of apo-CusA (PDB ID 3KO7). In panel B, the distances between P54 and A754 for the “binding” (PDB ID 7KF7), “extrusion” (PDB ID 7KF5), and “resting” (PDB ID 3KO7) protomers are 7.7 Å, 8.5 Å, and 7.6 Å, respectively. In panel C, these are superimpositions of the six “binding” protomers (left panel) and six “extrusion” protomers (middle panel) from the EEB (7KF7), EBB (7KF8), EEE (7KF5), and BBB (7KF6) structures, showing the comparison of the proton relay network at different conformational states. The left panel shows the overlay comparison of the one “binding” protomer of CusA EEB (light green), two “binding” protomers of CusA EBB (pink), and three binding protomers of CusA BBB (blue). The middle panel shows the comparison of the one “extrusion” protomer of CusA EBB (light green), two “extrusion” protomers of CusA EEB (pink), and three “extrusion” protomers of CusA EEE (blue). The superimpositions suggest that the six “binding” protomers are very similar in conformation (left panel). Likewise, the conformation of the six “extrusion” protomers are very similar to each other. The right panel displays the proton relay network of the “resting” protomer (bright green) of the X-ray structure of apo-CusA. Download FIG S2, JPG file, 2.3 MB. Copyright © 2021 Moseng et al. 2021 Moseng et al. https://creativecommons.org/licenses/by/4.0/ This content is distributed under the terms of the Creative Commons Attribution 4.0 International license.

10.1128/mBio.00452-21.5 TABLE S1 Cryo-EM data collection, processing, and refinement statistics. Download Table S1, PDF file, 0.10 MB. Copyright © 2021 Moseng et al. 2021 Moseng et al. https://creativecommons.org/licenses/by/4.0/ This content is distributed under the terms of the Creative Commons Attribution 4.0 International license.

In comparison with the “extrusion” conformers, the C-terminal end of the horizontal helix inside the periplasmic cleft of the “binding” protomer is found to tilt upward by ∼20^°^, which allows residue M672 to shift toward M573 and M623 to form the three-methionine metal binding site. Interestingly, an extra sphere-shaped density corresponding to the bound Cu(I) ion is found to coordinate the three methionine residues M573, M623, and M672 (Fig. 1D). The nearby conserved acidic residue E625 is also involved in binding Cu(I), stabilizing this ion via charge-charge interaction. With a combination of two “extrusion” protomers and one “binding” protomer, we designate the conformational state of this CusA trimer as the EEB form.

### Structure of trimeric CusA with one closed and two open periplasmic clefts.

**FIG 2:**
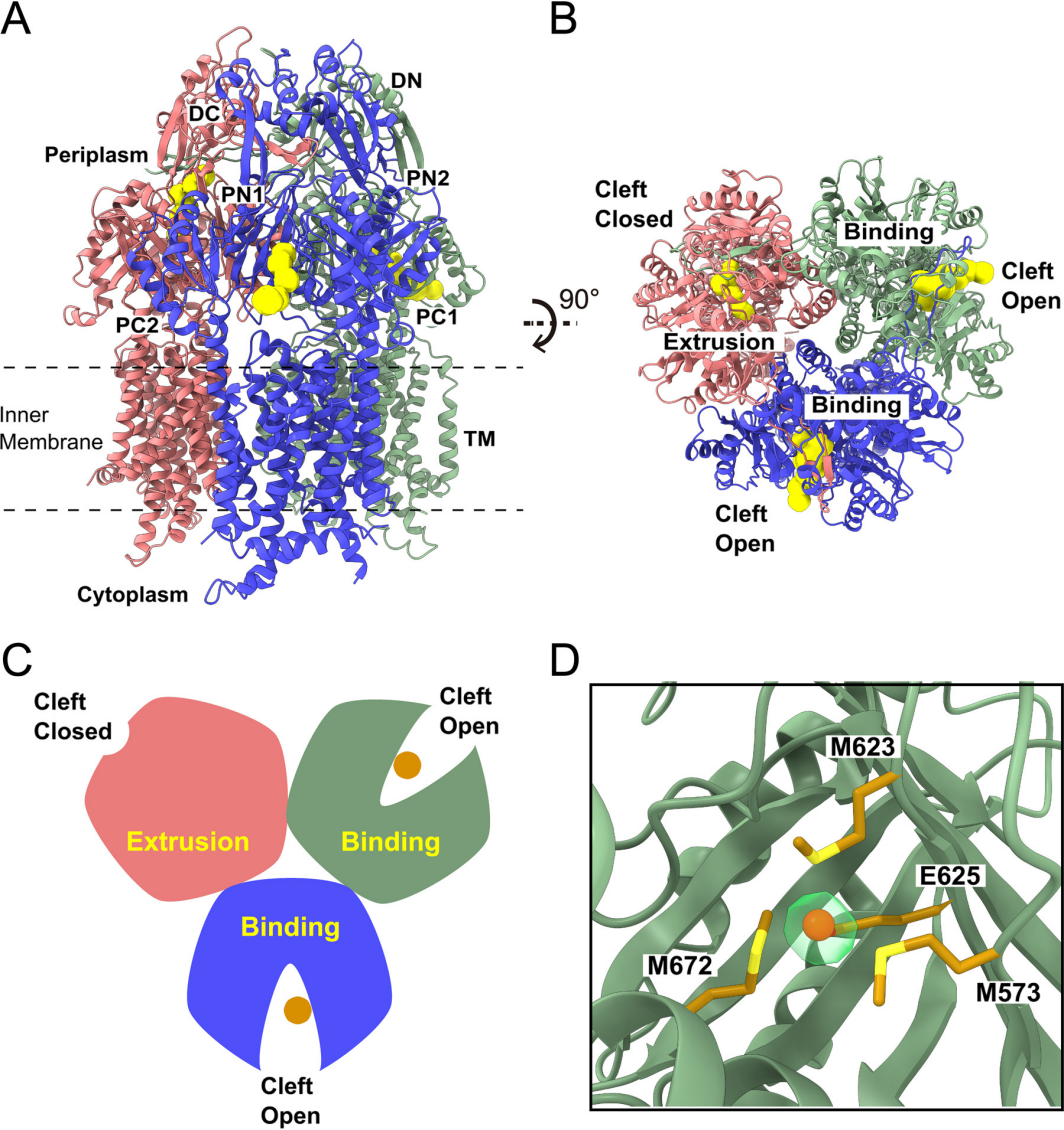
Cryo-EM structure of the CusA trimer in the EBB state. (A) Ribbon diagram of the CusA trimer with one closed cleft “extrusion” state protomer and two open cleft “binding” state protomers viewed from the membrane plane with the “extrusion” and “binding” channels shown in yellow. (B) Ribbon diagram of the EBB CusA trimer viewed from the top of the periplasmic domain illustrating the channels (colored yellow) formed in the “binding” and “extrusion” states of the CusA protomers. (C) A cartoon displaying the conformation of the CusA trimer in the EBB form viewed from the top of the periplasmic domain. In panels A, B, and C, the one “extrusion” and two “binding” protomers of CusA are colored pink, green, and blue, respectively. The bound Cu(I) ions within the two “binding” protomers are represented as dark orange circles. (D) The binding site of the Cu(I) ion (dark orange sphere) within the open periplasmic cleft of the “binding” protomer (green). The bound Cu(I) ion is coordinated by residues M573, M623, E625, and M672 (gold sticks). The density of bound Cu(I) is shown in transparent light green.

The second most abundant conformation we identified, with 43,395 single-particle counts in our cryo-EM data set, was the trimeric CusA conformation with one periplasmic cleft closed and two clefts open. We solved the cryo-EM structure of this CusA trimer to a resolution of 3.02 Å (Fig. 2, Fig. S1, and Table S1). For the CusA protomer with its periplasmic cleft closed, an “extrusion” channel extends perpendicular to the surface of the inner membrane and through the periplasmic domain. Again, this protomer depicts the “extrusion” state of the membrane protein. The conformations of the two protomers with the periplasmic cleft open are identical to each other, where a channel within the periplasmic cleft is found in each protomer (Fig. 2A to C). Each channel extends in parallel to the surface of the inner membrane, allowing the interior surface of the cleft in each protomer to be solvent exposed. These two protomers are in the “binding” conformation, which is nearly equivalent to the “binding” protomer described above. Similar to the “binding” protomer of the EEB trimer, the C-terminal end of the horizontal helix inside the periplasmic cleft is observed to tilt upward by ∼20^°^, leading to the formation of the M573-M623-M672 methionine binding site in these two protomers. A Cu(I) ion anchors at the center of the three-methionine binding site of each “binding” protomer, utilizing residues M573, M623, M672, and E625 to secure Cu(I) binding (Fig. 2D). As the structure of this trimeric CusA pump is characterized by one “extrusion” and two “binding” protomers, we label the conformational state of this trimer as the EBB form.

### Structure of trimeric CusA with three closed periplasmic clefts.

**FIG 3:**
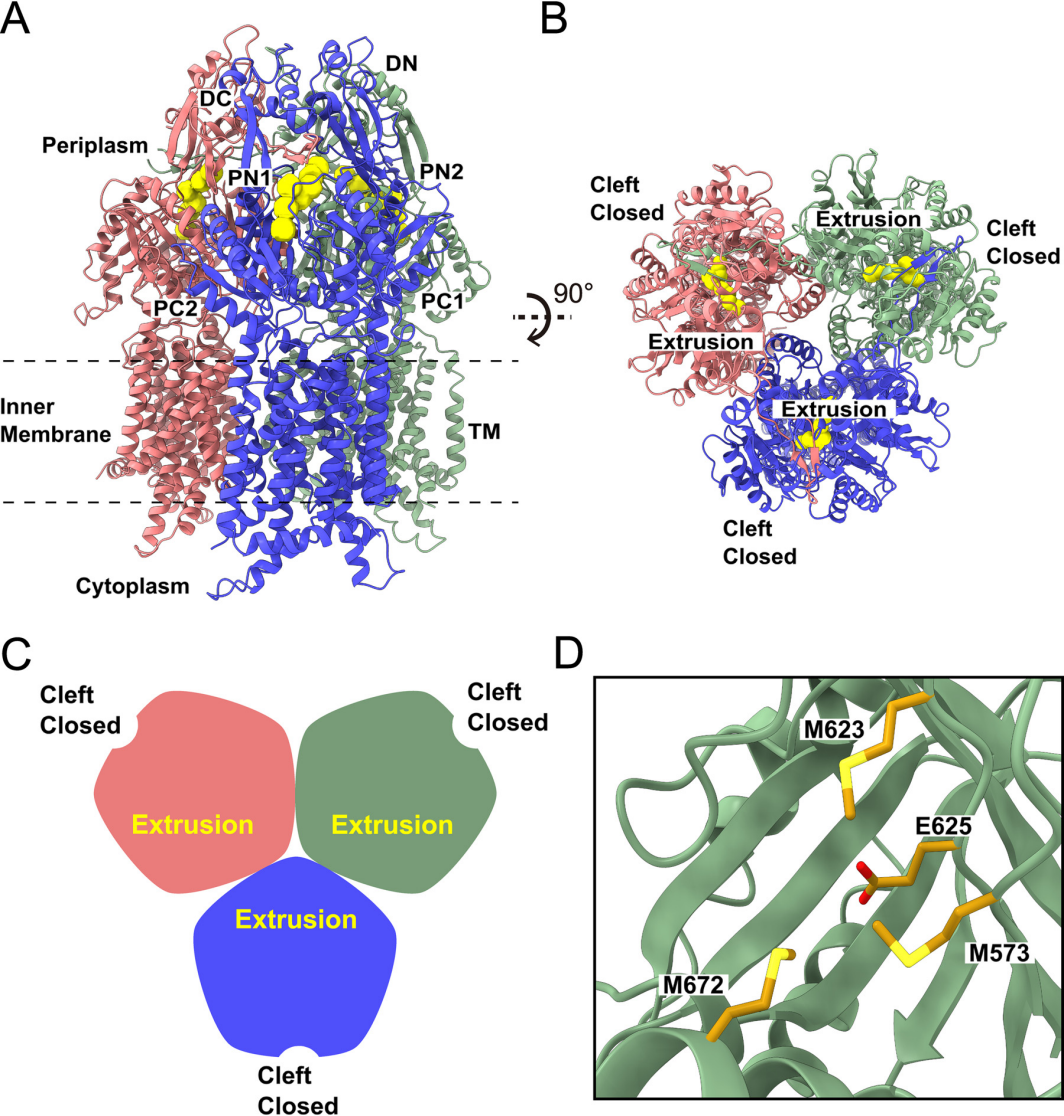
Cryo-EM structure of the CusA trimer in the EEE state. (A) Ribbon diagram of the CusA trimer with three closed cleft “extrusion” state protomers viewed from the membrane plane with the “extrusion” channels shown in yellow. (B) Ribbon diagram of the EEE CusA trimer viewed from the top of the periplasmic domain illustrating the channels (colored yellow) formed in the “extrusion” states of the CusA protomers. (C) A cartoon displaying the conformation of the CusA trimer in the EEE form viewed from the top of the periplasmic domain. In panels A, B, and C, the three “extrusion” protomers of CusA are colored pink, green, and blue, respectively. (D) The Cu(I) binding site. No extra density representing bound Cu(I) is found within closed periplasmic cleft of this “extrusion” protomer (green). Residues M573, M623, E625, and M672 responsible for forming the Cu(I) binding site are in gold sticks.

The third most populated conformation of trimeric CusA in our cryo-EM sample has all three periplasmic clefts closed. We obtained 21,108 single-particle images for this conformation in our cryo-EM data set. The cryo-EM structure of this CusA trimer was solved to a resolution of 3.20 Å (Fig. 3, Fig. S1, and Table S1). Interestingly, the conformations of these three protomers are identical, presenting a symmetric, trimeric structure. The conformation of these three protomers is very similar to the conformation of the “extrusion” protomers of the EEB and EBB structures, with an “extrusion” channel observed to extend vertically through the periplasmic domain of each protomer with respect to the membrane surface (Fig. 3A and B). We, therefore, classify the conformational state of this symmetric trimer as the EEE form of CusA.

### Structure of trimeric CusA with three open periplasmic clefts.

**FIG 4:**
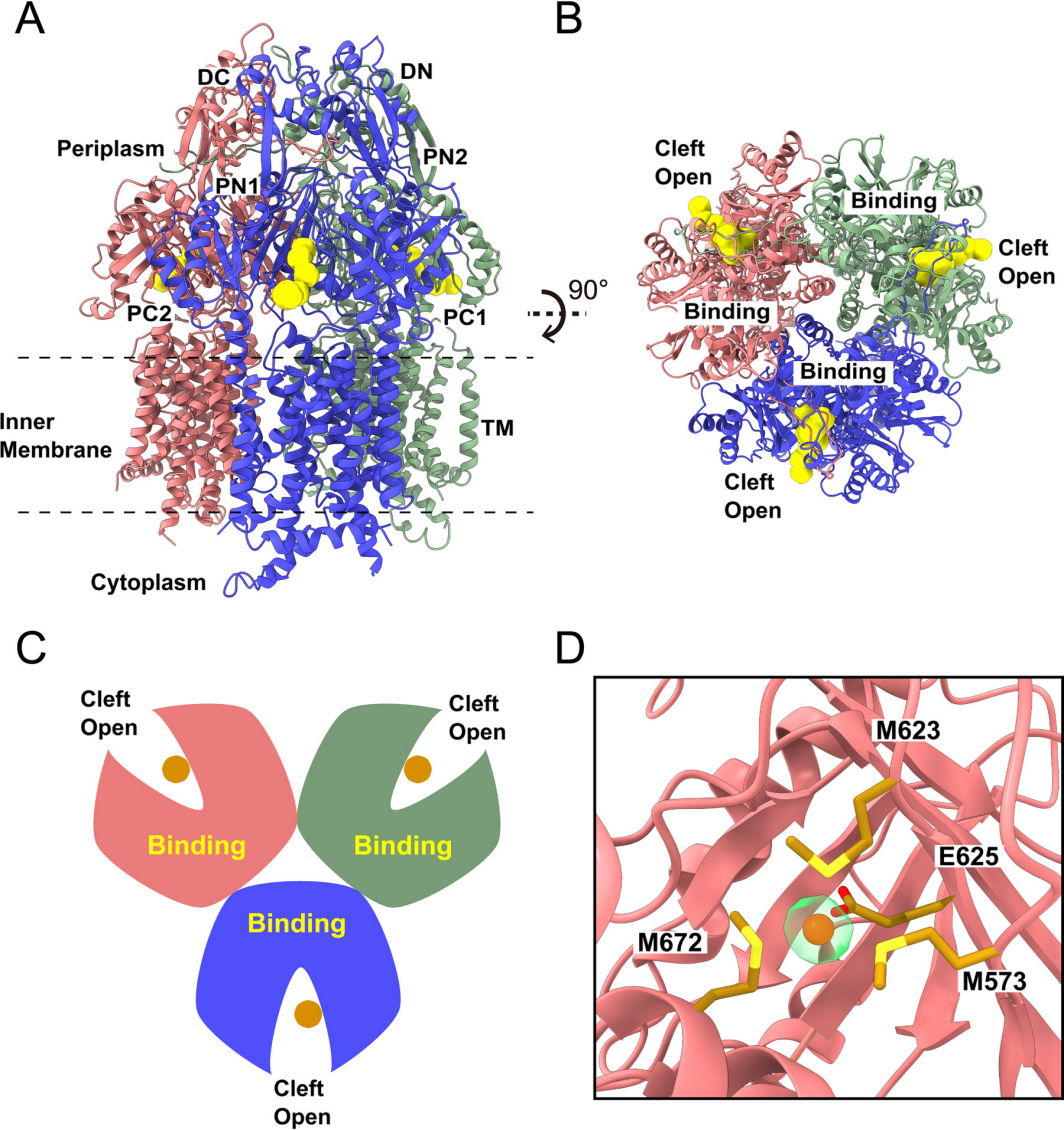
Cryo-EM structure of the CusA trimer in the BBB state. (A) Ribbon diagram of the CusA trimer with three open cleft “binding” state protomers viewed from the membrane plane with the “binding” channels shown in yellow. (B) Ribbon diagram of the BBB CusA trimer viewed from the top of the periplasmic domain illustrating the channels (colored yellow) formed in the “binding” states of the CusA protomers. (C) A cartoon displaying the conformation of the CusA trimer in the BBB form viewed from the top of the periplasmic domain. In panels A, B, and C, the three “binding” protomers of CusA are colored pink, green, and blue, respectively. (D) The binding site of the Cu(I) ion (dark orange sphere) within the open periplasmic cleft of the “binding” protomer (pink). The bound Cu(I) ion is coordinated by residues M573, M623, E625, and M672 (gold sticks). The density of bound Cu(I) is shown in transparent light green.

The least abundant conformation of trimeric CusA has three protomer clefts open. We obtained 13,304 single-particle images for this conformation in our cryo-EM sample data set. We solved the cryo-EM structure of this CusA trimer to a resolution of 3.40 Å (Fig. 4, Fig. S1, and Table S1). In this trimeric CusA structure, the conformations of the three protomers are mostly equivalent, also presenting a symmetric, trimeric configuration. A “binding” channel is found in the periplasmic cleft of each protomer, indicating that the three protomers are in their “binding” conformation (Fig. 4A to C). In addition, it is observed that a bound Cu(I) is anchored at the three-methionine binding site of each protomer, anchored by residues M573, M623, M672, and E625 (Fig. 4D). As the three CusA protomers are in their “binding” form, we label this trimer as the BBB conformation of the CusA pump.

### The proton relay network.

**FIG 5:**
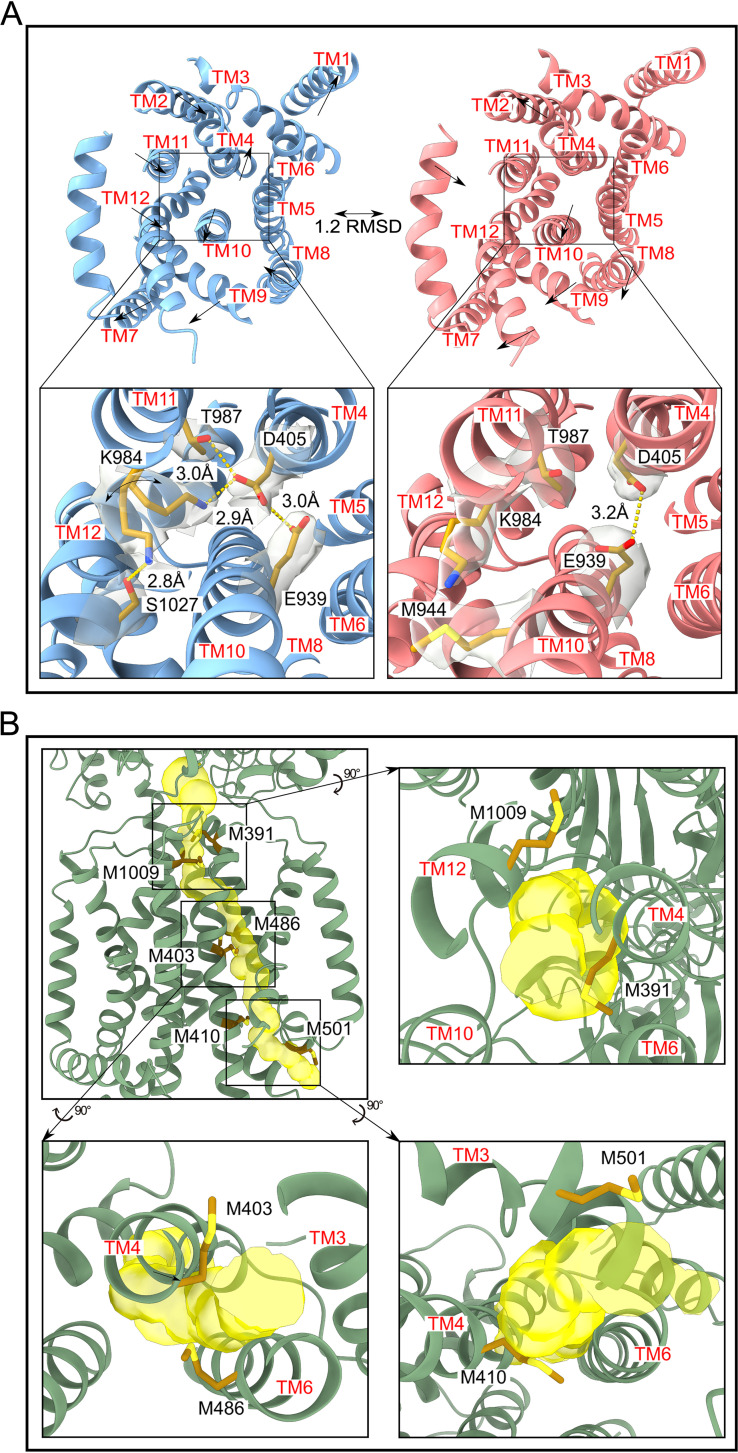
The proton relay and methionine relay networks. (A) The proton relay network of the CusA heavy-metal efflux pump. (Left) The binding state of CusA (light blue). In this conformational state, the “proton sweeper” K984 was found to have two alternate rotamer conformations. One rotamer forms a hydrogen bond (yellow dashed line) with D405. D405 also forms hydrogen bonds with E939 and T987. The other rotamer of K984 is positioned away from D405 and forms a hydrogen bond with S1027. Black arrows represent the motion of the TM helices when they shift to the extrusion state. The densities of residue side chains (D405, K984, E939, and T987), which form the proton relay network are represented as a gray transparent surface. (Right) The extrusion state of CusA (light pink). In this conformation, K984 is swung away from D405 oriented to participate in a dipole-dipole interaction with M944. Superimposition of the CusA TM domain of the binding and extrusion states gives rise to an r.m.s.d. of 1.2 Å. (B) The methionine relay network of the CusA heavy-metal efflux pump. (Top left) Ribbon diagram of the binding state protomer of CusA with the channel for metal ion transport (yellow) and methionine residues (dark gold sticks) participating in metal transport viewed from the transmembrane region of the “binding” protomer of the EEB structure with the channel spanning from the cytosol into the periplasmic cleft. Black arrows indicate magnification and rotation to view the methionine pairs. (Top right) The methionine pair M1009-M391 near the entrance into the periplasmic cleft. (Bottom left) The methionine pair M486-M403 positioned in the middle of the transmembrane domain. (Bottom right) The methionine pair M501-M410 with side chains oriented away from each other near the entryway into the transmembrane domain from the cytosol.

It has been well established that the proton motive force (PMF) of the cell powers RND efflux pumps to extrude drugs from the periplasmic domain. In the transmembrane domain of CusA, the conserved residues D405, E939, and K984 of the HAE-RND efflux pump family are necessary for the efflux of metal ions (13). The density of our cryo-EM maps unambiguously depicts the side chain positions of these conserved amino acids, allowing us to elucidate the mechanism of proton transfer within this proton relay network. Data have shown that a point mutation at D405, E939, or K984 of CusA renders this pump unable to transport metal ions across the membrane, suggesting that the proton relay network may be necessary for the transition between different conformational states (13). The cryo-EM structures of CusA clearly identify two distinct protomers, labeled as either “binding” or “extrusion” conformers, with distinct structural differences between the two states. When compared to the “binding” protomer, the “extrusion” protomer displays a rearrangement of side chains of the proton relay residues as well as side chain residue shifts within the transmembrane helices TM4, TM7, TM8, TM9, TM10, TM11, and TM12 (Fig. 5A). Coupled with the movement of closing the periplasmic cleft in the “extrusion” state, TM8 is found to shift toward the core by as much as 10 Å. Other transmembrane helices, TM7, TM9, and TM10, also shift horizontally by 4 Å, mimicking the motion of TM8. Interestingly, TM1 and TM4 undergo a 3-Å upward shift with respect to the inner membrane surface. The net result is that all of these transmembrane helices rearrange in a twisting motion constricting the transmembrane domain.

The movements of these transmembrane helices are accompanied by the reorientation of the side chain of the proton sweeper K984 toward the acidic residue D405 (Fig. 5A). In each “binding” conformer, D405 appears to form hydrogen bonds with T987, E939, and K984. Interestingly, the structure of the EEB CusA trimer indicates that there is an alternate conformation of the proton sweeper K984 within the “binding” conformer (Fig. 5A). The side chain of K984 sweeps away from D405 and shifts toward the cytoplasm. This alternate side chain orientation also creates a new hydrogen bond with S1027. In this manner, the proton can be effectively passed from D405 to K984, which then sweeps and passes the proton to S1027, coupling with the opening of the periplasmic cleft to bind Cu(I). To advance the transport cycle, the CusA protomer may shift to the “extrusion” form as observed in the cryo-EM structures. At this state, the transmembrane helices shift in conformation to constrict the transmembrane domain. The side chain nitrogen of K984 is found to move away from S1027, interacting with M944 to form a dipole-dipole interaction and potentially stabilize this transient state. Additionally, in the “extrusion” state, E939 forms a hydrogen bond with D405 that may be necessary to reset the side chains of the proton relay network residues for the subsequent cycle (Fig. 5A). Taken together, these cryo-EM structures illuminate the role of the proton sweeper K984 in the proton relay network. It appears that K984 plays a major role in transferring protons from D405 to S1027 to advance this transfer process.

### The transmembrane methionine relay channel.

In the transmembrane region of each CusA protomer, six methionines align to form three pairs. These three methionine pairs are M410-M501, M403-M486, and M391-M1009, which line up with the methionine triad M573-M623-M672 and another methionine pair, M271-M755, within the periplasmic domain, to assemble the methionine relay network (13). Previously, it has been discovered that CusA is capable of transporting metal ions from the cytosol via these methionines. A single point mutation within this methionine network is able to completely abolish metal transport. The ability of RND transporters to pick up substrates from the cytoplasm has also been seen from the ZneA (14), CzcA (19), and AcrD (20) pumps. It appears that the three methionine pairs located at the transmembrane domain are made up of residues from TM4, TM6, and TM12. Therefore, it is likely that these transmembrane helices help shuttle metal ions across the transmembrane.

The three methionine pairs located within the transmembrane regions of CusA display significantly different conformations between the cryo-EM structures of the “extrusion” and “binding” states of each protomer. For example, the distances between the sulfur atoms of M410-M501, M403-M486, and M391-M1009 are 5.4 Å, 5.9 Å, and 5.7 Å in the “extrusion” state of CusA, respectively. These distances become 12.1 Å, 4.4 Å, and 11.4 Å in the “binding” state (Fig. 5B). As TM4 residues participate in creating both the proton relay network (e.g., D405) and the methionine relay network (e.g., M391, M403, and M410), this transmembrane helix is likely critical for coupling the processes of proton import and metal ion export. It appears that proton transfer via D405 may trigger a conformation change of TM4, which in turn initiates the metal ion transport process.

In each “binding” conformer of CusA, a channel spanning the entire transmembrane region up to the periplasmic domain is observed. This channel passes through the three methionine pairs in the transmembrane domain, presumably relaying the metal ion to shuttle across the membrane. However, this channel is absent in each “extrusion” conformer. Based on the cryo-EM structures, the metal ion from the cytosol would first encounter the M410-M501 pair that extends into the cytosol, forming the entrance of the methionine relay channel formed between TM3, TM4, and TM6 at the transmembrane-cytosolic interface. In the “binding” state, the S-methyl thioether side chain of M410 and M501 appear to face away from one another with the M501 side chain exposed to the cytosol, seemingly allowing the metal ion to enter this channel (Fig. 5B). In the “extrusion” state, this entrance is closed, largely due to the shift in locations of TM5 and TM4, which reorient the side chains of M410 and M501 toward one another, constricting this opening. The next methionine pair in the channel is M403-M486, located in the middle of the transmembrane domain between TM4 and TM6. At this area, Cu(I) or Ag(I) ions could pass between TM4 and TM6 and up to the third methionine pair M391-M1009 located near the outer leaflet surface of the cytoplasmic membrane. Interestingly, the S-methyl thioether side chains of M391 and M1009 are oriented away from each other in each “binding” conformer (Fig. 5B). This conformation allows this transmembrane channel to completely open to the periplasm. When compared with the “binding” protomers, the conformation of each “extrusion” protomer depicts that the transmembrane helices TM4, TM6, TM10, and TM12 move toward one another, constricting the channel and reorienting the side chains of M391 and M1009 to effectually shut the opening of the channel facing the periplasm.

### Molecular simulations of CusA cryo-EM structures in both trimeric and monomeric state.

We assessed the stability of the cryo-EM structures using molecular dynamics (MD) simulations (Table S2). Here, we calculate the stability of the protein using r.m.s.d. in all structures obtained with cryo-EM over the first 200 ns of the simulation. Our data indicate that the secondary structure obtained are stable (Cα r.m.s.d. < 3.0 Å) throughout the simulation (Fig. S3A and B). The root mean square fluctuation (r.m.s.f.) of residues are similar in both the extruded and bound states. By comparing trimeric simulation to the monomeric simulation, the r.m.s.f. value differs only between residues 220 to 230, which are located at the trimer interface. However, the r.m.s.f. between “binding” and “extrusion” conformations are similar (Fig. S3B and C). This suggests that each monomer works independently and validates the quality of the structure obtained, which provides a platform for a subsequent study in this paper.

10.1128/mBio.00452-21.3 FIG S3 Cα r.m.s.d. and residual r.m.s.f. (A) Cα r.m.s.d. of the cryo-EM structures. Calculated Cα r.m.s.d. of the first 200-ns simulation of the trimeric cryo-EM structures with three Cu(I)-bound subunits (BBB), two Cu(I)-bound subunits, and one extrusion state (EBB), one Cu(I)-bound state (EEB), and all three subunits in the extrusion state (EEE). The three colors indicate the three different repeats. The shaded region shows the running average every 1 ns. (B) Residual r.m.s.f. and secondary structure retention of the cryo-EM structures. Calculated residue r.m.s.f. of the first 200-ns simulation of the trimeric cryo-EM structures with three Cu(I)-bound subunits (BBB), two Cu(I)-bound subunits, and one extrusion state (EBB), one Cu(I)-bound state (EEB), and all three subunits in the extrusion state (EEE). The secondary structure analysis was sampled every 2 ns. The shaded region indicates the standard deviation around the mean of three repeats. (C) Residual r.m.s.f. and secondary structure retention of the monomeric structures. Calculated residue r.m.s.f. of 500-ns simulation of the monomeric structures with bound configuration in the presence and absence of copper (B-Cu and B-Apo) and the extruded state in the presence and absence of copper (E-Cu and E-Apo). The secondary structure analysis was sampled every 5 ns. The shaded region indicates the standard deviation around the mean of three repeats. Download FIG S3, JPG file, 1.7 MB. Copyright © 2021 Moseng et al. 2021 Moseng et al. https://creativecommons.org/licenses/by/4.0/ This content is distributed under the terms of the Creative Commons Attribution 4.0 International license.

10.1128/mBio.00452-21.6 TABLE S2 Simulations conditions and set-up. Download Table S2, PDF file, 0.04 MB. Copyright © 2021 Moseng et al. 2021 Moseng et al. https://creativecommons.org/licenses/by/4.0/ This content is distributed under the terms of the Creative Commons Attribution 4.0 International license.

### Cu(I)-dependent conformational change.

**FIG 6:**
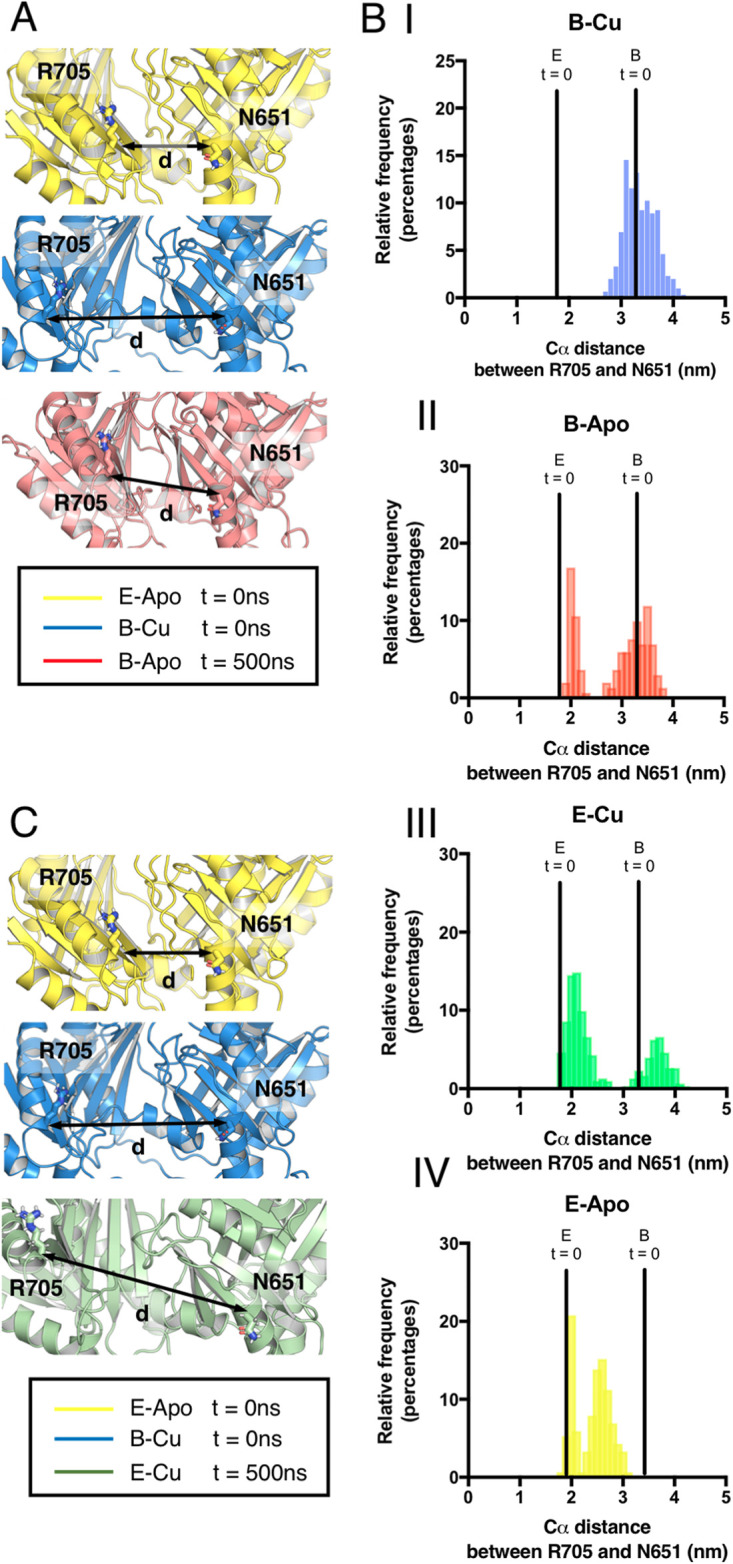
Conformational change of a CusA monomer. (A) The final frame of a monomeric CusA 500-ns simulation starting from the “binding” state (red). This is structurally aligned with cryo-EM structures of the “extrusion” state (yellow) and “binding” state (blue). The spheres show the positions of the Cα atoms of N651 and R705. (B) Distribution of distances between Cα atoms of R705 and N651 over the last 100 ns of 500-ns simulations from the “extrusion” state with Cu-bound (E-Cu [panel III]), no Cu (E-Apo [panel IV]), “binding” state without Cu(I) (B-Apo [panel II]) and “binding” state with Cu(I) (B-Cu [panel I]). (C) The final frame of a monomeric CusA 500-ns simulation starting from an “extrusion” state with a Cu(I) ion docked into the binding site. This is structurally aligned with cryo-EM structures of the “extrusion” state (yellow) and “binding” state (blue). The spheres show the positions of the Cα atoms of N651 and R705.

To assess the role of Cu(I) in the conformational transitions of the periplasmic domain, we performed simulations of the “extrusion” and “binding” protomers of CusA, with and without Cu(I) added. Based on the major differences between the two states, the distance between the Cα atoms of N651 and R705 was used as a metric of calculating conformational change. Simulations of the “extrusion” state [−Cu(I)] and the “binding” state [+Cu(I)] of CusA resulted in a consistent Cα distance between these two residues measured throughout the simulations (Fig. 6A and B). In contrast, removal of Cu(I) from the “binding” state resulted in CusA transitioning toward the “extrusion” state in one of the three simulations performed (Fig. 6A and B). At the end state of this repeat, the Cα distance between N651 and R701 was equivalent to that of the structure of the “extrusion” state (Fig. 6A and B and Fig. S4A).

10.1128/mBio.00452-21.4 FIG S4 Conformational change of CusA. (A) Conformational change between bound and extruded state structure in a monomer. Distances between Cα atoms of R705 and N651 over 500-ns simulations from the “extrusion” state without Cu(I) (E-Apo), “extrusion” state with Cu(I) (E-Cu), “binding” state without Cu(I) (B-Apo), and “binding” state with Cu(I) (B-Cu). The three colors indicate the three different repeats. (B) Conformational change of the CusA trimer in a modeled E. coli cell membrane. Distribution of distance between Cα R705 and Cα N651 over the last 100 ns of 450-ns simulations from the “binding” state with bound Cu(I) (BBB-Cu [A]), “binding” state without Cu(I) (BBB-Apo [B]), “extrusion” state with bound Cu(I) (EEE-Cu [ C]) and “extrusion” state without Cu(I) (EEE-Cu [D]). The distributions were taken from 3 monomers × 3 simulations, resulting in 909 data points per each analysis. (C) Conformational change of the CusA trimer. Measurement of distance between Cα R705 and Cα N651 over 450-ns simulations from the “binding” state with bound Cu(I) (BBB Cu), “binding” state without Cu(I) (BBB Apo), “extrusion” state with bound Cu(I) (EEE Cu), and “extrusion” state without Cu(I) (EEE Apo). The three colors indicate the three different repeats. The shaded region shows the running average every 1 ns. Download FIG S4, JPG file, 1.9 MB. Copyright © 2021 Moseng et al. 2021 Moseng et al. https://creativecommons.org/licenses/by/4.0/ This content is distributed under the terms of the Creative Commons Attribution 4.0 International license.

Evaluation of this simulation suggests that the absence of Cu(I) leads to instability in the coordinating residues D671 and M672. D671 establishes a salt bridge with K678, and this interaction rigidly pulls the PC2 subdomain toward PC1. This transition is further supported by a hydrophobic collapse of the adjacent residues, including the helix that connects PC1 to PC2 (see Movie S1 in the supplemental material).

10.1128/mBio.00452-21.7 MOVIE S1 Transition from “binding” to “extrusion.” Download Movie S1, MP4 file, 7.4 MB. Copyright © 2021 Moseng et al. 2021 Moseng et al. https://creativecommons.org/licenses/by/4.0/ This content is distributed under the terms of the Creative Commons Attribution 4.0 International license.

Conversely, the addition of Cu(I) to the “extrusion” conformation leads to a separation of R705 and N651 in one of the three simulation repeats, as the structure transitions toward a state equivalent to that of the bound structure (Fig. 6C and D). The dynamics of the motions are similar to a reverse of what is described for the removal of Cu(I) from the “binding” state (Movie S2). These simulations suggest that the conformational changes observed for CusA are driven by Cu(I).

10.1128/mBio.00452-21.8 MOVIE S2 Transition from “extrusion” to “binding.” Download Movie S2, MP4 file, 24.7 MB. Copyright © 2021 Moseng et al. 2021 Moseng et al. https://creativecommons.org/licenses/by/4.0/ This content is distributed under the terms of the Creative Commons Attribution 4.0 International license.

### The role of a trimeric structure in a Cu(I)-dependent conformational change.

To observe whether the transition in a monomer exists in a trimer, we conducted three repeats of 450-ns simulations on both the trimeric “extrusion” state (EEE) and “binding” state (BBB) with and without Cu(I). For both the EEE state and the BBB state without Cu(I) obtained from the cryo-EM structure, the simulations did not deviate from the starting structures (Fig. S4B). Unlike the monomeric simulations, removal of Cu(I) from the bound state did not result in any structural rearrangement of the PC1 and PC2 domains (Fig. S4B), indicating that the trimeric oligomerization is capable of stabilizing the structures of the BBB form both in the absence and presence of Cu(I).

To test the dynamics of the “extrusion” state of CusA, we added Cu(I) to the binding site in all three subunits of the EEE trimer. In these simulations, we observed transitions from the “extrusion” state toward the “binding” state (Fig. S4B). This transition is similar to that observed with the monomer simulation and occurs independent of the adjacent subunits (Fig. S4C). CusA functions as a trimer, and this trimeric oligomerization is probably capable of stabilizing each conformational state of the pump.

### Water wires mediate proton transport through the transmembrane domain of CusA.

**FIG 7:**
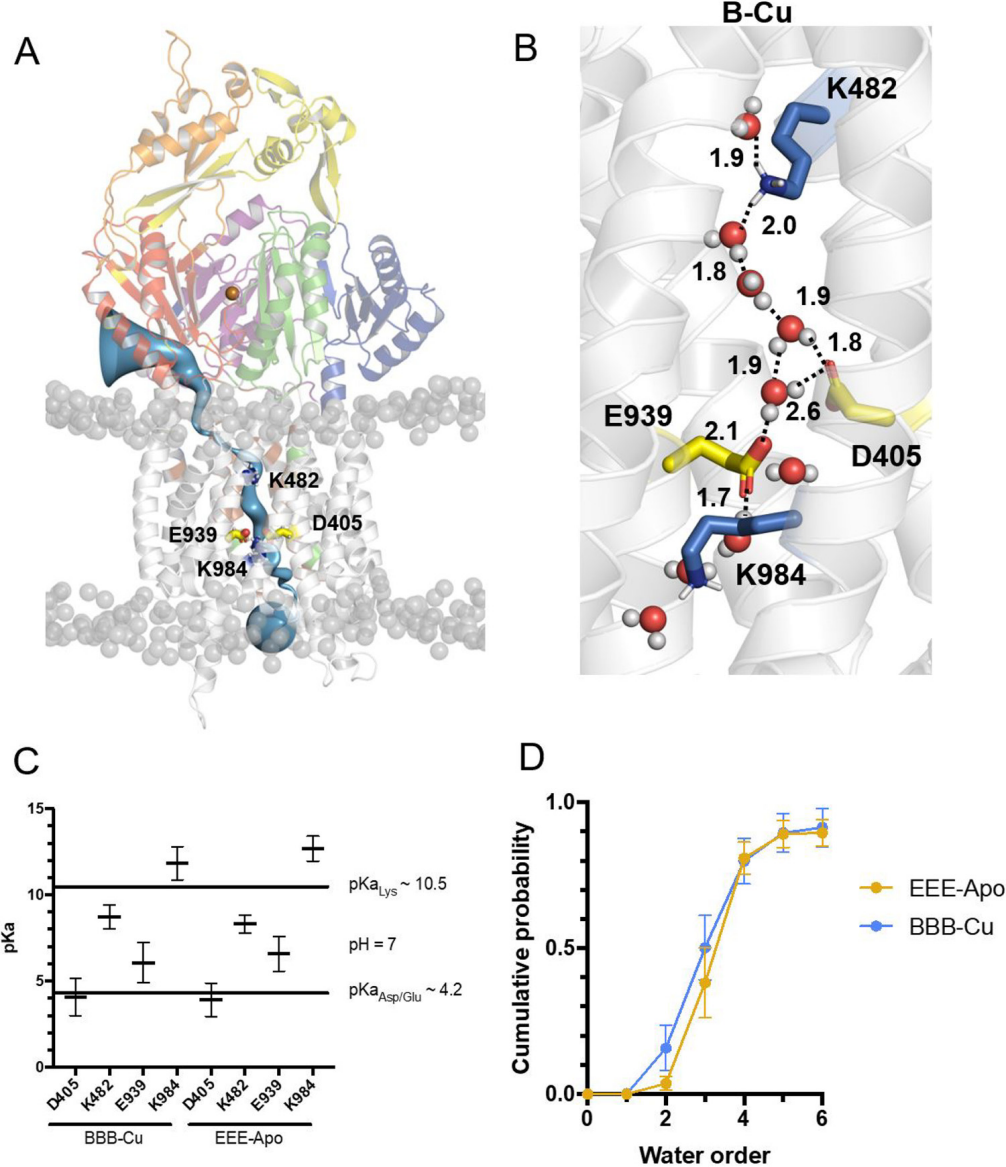
Proton permeation path through the CusA protein. (A) An analysis of the pore profile along CusA (blue) with protonatable residues highlighted in red for acidic residues (D405 and E939) and blue for basic residues (K482 and K984). Pore-lining residues are highlighted in green for polar residues and brown for hydrophobic residues. Different subdomains are colored in purple (PC2), blue (PC1), orange (DC), yellow (DN), green (PN1), and red (PN2). (B) A path of proton permeation across the membrane via a water wire from basic residues (blue) to acidic residues (yellow) based on the “extrusion” state of a protomer from the EEE trimer. The structure displays a solvated pore after 20-ns equilibration with Cα restrained. Putative hydrogen bonds are shown as black dashes with distances in angstroms. (C) Calculated pK_a_ values of the four acidic and basic residues in the transmembrane region of the protein from the last 100 ns of the 450-ns simulation in the trimeric CusA pump with and without Cu(I). Data are shown for three repeats of the three subunits. Error bars display the standard errors of the means (*n* = 9). (D) Calculated number of water molecules involved in the water wire from the last 100 ns of the 450-ns simulation of trimeric CusA with (blue) and without (yellow) Cu(I). Data are shown for three repeats of the three subunits only in the subunit when water molecules are present in the pore. Error bars display the standard errors of the means (*n* = 8).

In addition to evaluating conformational changes of the periplasmic domain, we also investigated the solvent accessibility of the CusA structures in lipid membranes, with the aim of proposing a putative proton transfer pathway through CusA that would be used to drive copper transport. In both the monomeric (E and B states) and trimeric (EEE and BBB states) structures, we observe a membrane-spanning water wire within the transmembrane (TM) domains, where the water wire directly connects the proton relay residues D405, E939, and K984 to the conserved lysine K482 (Fig. 7A and B and Movie S3). To evaluate the path of the water wire, and therefore, by inference, proton transfer, we calculated the pK_a_ values of all acidic and basic residues present in the transmembrane region from the last 100 ns of three 450-ns trajectories of the trimeric structures. The analysis indicates that the pK_a_ values of two pore-lining side chains, K482 and E939, are nearly 7, suggesting that both residues are proton labile (Fig. 7C). As both residues are at either end of the permeation pathway, we calculated the number of water molecules that were found to bridge the two side chains. The most likely number of bridging waters between K482 and E939 was four (Fig. 7D). On the basis of this finding, we suggest that a proton could start on the periplasmic side of the membrane and hop from K482 via the bound water to the proton relay triad D405-E939-K984, and eventually be delivered to the cytoplasmic side through the flipping in conformation of the side chain of the proton sweeper K984.

10.1128/mBio.00452-21.9 MOVIE S3 The membrane-spanning water wire. Download Movie S3, MP4 file, 11.8 MB. Copyright © 2021 Moseng et al. 2021 Moseng et al. https://creativecommons.org/licenses/by/4.0/ This content is distributed under the terms of the Creative Commons Attribution 4.0 International license.

## DISCUSSION

Here we have defined cryo-EM structures of the CusA metal ion efflux pump in the presence of Cu(I). These structural data allow us to depict four distinct structures of the CusA trimer within a single cryo-EM sample. We revealed that three CusA molecules can assemble as symmetric trimers as indicated by the EEE and BBB trimeric structures, where all three protomers are in identical “extrusion” (E) or “binding” (B) conformations within the trimer. In addition, we detected that the three CusA molecules can assemble as asymmetric trimers as suggested by the EEB and EBB structures. The EEB trimer delineates the assembly of two “extrusion” protomers and one “binding” protomer, whereas the EBB trimer contains one “extrusion” and two “binding” protomers within the CusA trimer.

We clearly observed from the cryo-EM structures that different CusA protomers within the trimer are able to simultaneously accommodate Cu(I). Each “binding” protomer of CusA from the EEB, EBB, and BBB conformational assemblies is occupied by a Cu(I) ion, where the metal ion is coordinated and secured by the familiar three-methionine binding site composed of M573, M623, and M672. The conserved negatively charged residue E625 also contributes to neutralize the formal positive charge of the bound Cu(I) ion. This observation highlights a phenomenon that individual protomers within the CusA trimer are capable of independently binding and exporting metal ions. Our experimental data are in line with results from MD simulations that each CusA protomer works independently. In addition, our cryo-EM and computational findings are in good agreement with the recent study of the C. jejuni CmeB HAE-RND-type multidrug efflux pump, where single-molecule FRET imaging indicated that the three CmeB protomers within the trimer can simultaneously bind and export substrates (10). Each CmeB subunit can undergo uncoordinated conformational transitions and function independently.

We believe that CusA is capable of picking up metal ions from both the periplasm (via the periplasmic cleft) and cytoplasm (via the three methionine pairs at the transmembrane region). As soon as the metal ion arrives at the three-methionine binding site (M573-M623-M672) deep inside the periplasmic cleft of CusA, this bound metal ion could then be released to the nearest methionine pair (M271-M755) directly above the three-methionine binding site. Subsequently, the ion could exit the CusA pump via the extrusion channel and eventually reach the CusB and CusC channels for final extrusion from the bacterial cell.

Single-molecule FRET imaging of CmeB efflux revealed that there are at least four distinct conformational states of the CmeB protomer transitioning within the substrate transport cycle (10). Previously, X-ray crystallography discovered that the CusA protomers take a “resting” state in the absence of Cu(I) or Ag(I) ions, where the three periplasmic clefts are closed. However, these protomers acquire a “binding” conformation with an open periplasmic cleft in the presence of Cu(I) or Ag(I) (13). In the present work, our cryo-EM study allowed us to observe that the CusA protomers are capable of forming an “extrusion” state with the periplasmic cleft closed and a “binding” state with the periplasmic cleft open in the presence of Cu(I). Based on our findings, it is likely that the CusA pump, belonging to the HME-RND family, may go through a simpler transport cycle when compared to that of the CmeB HAE-RND pump. This may be because only one metal ion binding site has been observed within the periplasmic cleft of CusA, whereas multiple binding sites, such as at the entrance, proximal, and distal drug binding sites, have been seen with the HAE-RND efflux pumps (3, 5, 21). Indeed, the secondary structural elements within the periplasmic clefts of HME-RND and HAE-RND efflux pumps are very distinct from each other. For example, deep inside the clefts of AcrB (21), CmeB (10), MtrD (9), and AdeB (11), there are two conserved flexible loops which are functionally important for these pumps. In each of these multidrug efflux pumps, the F-loop forms part of the proximal drug binding site and connects this proximal site to the cleft entrance, whereas the G-loop compartmentalizes the proximal and distal drug binding sites. In CusA, there is no G-loop in the structure. The space that is normally occupied by the G-loop of a HAE-RND pump becomes a free cavity in CusA. In addition, residues corresponding to the F-loop of a HAE-RND pump compile to form the horizontal helix which is critical for Cu(I) binding in CusA.

**FIG 8:**
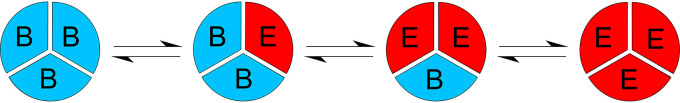
Proposed model of heavy-metal efflux mechanism. During heavy-metal export, each protomer of the trimeric CusA pump autonomously undergoes a sequence of conformational transitions. This schematic diagram indicates that each protomer within the CusA trimer is able to independently go through conformational transitions, leading to the extrusion of metal ions (B, “binding” state; E, “extrusion” state).

In the absence of Cu(I), the CusA protomers prefer the “resting” conformation, where the three periplasmic clefts are closed within the trimer. In the presence of Cu(I), we observed only the “binding” state with the periplasmic cleft open and the “extrusion” state with the periplasmic cleft closed. It is possible that the CusA pump can easily continue to advance the transport cycle from the “binding” to “extrusion” conformations by coupling with proton transfer via the proton relay network coupled with the proton wire. Our data allow us to propose a simple model for the transport mechanism of the CusA HME-RND pump (Fig. 8), where the CusA protomers can independently and uncoordinatedly function to export metal ion by progressing from the “binding” state to the “extrusion” state within the transport cycle.

It should be noted that CusA works with CusB and CusC to form the CusA_3_-CusB_6_-CusC_3_ tripartite efflux assemblage (15) to export Cu(I) and Ag(I) ions, and the contacts between the CusA pump and CusB adaptor could influence the states of the CusA monomers, presumably tuning the efflux system to become more efficient. Indeed, a stopped-flow assay suggested that the reconstituted CusA-CusB proteoliposomes are two times more active than those proteoliposomes containing CusA only for metal transport (22).

## MATERIALS AND METHODS

### Expression and purification of CusA.

The *cusA* gene, encoding the CusA heavy-metal efflux pump, from E. coli was cloned into the pET15b expression vector in frame with a 6×His tag at the C terminus to generate the pET15bΩ*cusA* plasmid. The CusA protein was overexpressed in E. coli BL21(DE3)Δ*acrB*/pET15bΩ*cusA* cells, which harbor a deletion in the chromosomal *acrB* gene. Cells were grown in 6 liters of LB medium with 100 μg/ml ampicillin at 37°C. When the optical density at 600 nm (OD_600_) reached 0.4, the culture was treated with 0.2 mM isopropyl β-d-1-thiogalactopyranoside (IPTG) to induce CusA expression. Cells were then harvested within 3 h of induction. The collected bacteria were resuspended in low salt buffer containing 20 mM HEPES-NaOH (pH 7.0), 10% glycerol, and 1 mM phenylmethanesulfonyl fluoride (PMSF) and then disrupted with a French pressure cell. The membrane fraction was collected and washed twice with 20 mM HEPES-NaOH buffer (pH 7.0) containing 1 mM PMSF. The membrane protein was then solubilized in 1% (wt/vol) (Cymal-6). Insoluble material was removed by ultracentrifugation at 100,000 × *g*. The extracted protein was then purified with an Ni^2+^ affinity column. The purity of the CusA protein (∼95%) was judged using sodium dodecyl sulfate-polyacrylamide gel electrophoresis (SDS-PAGE) stained with Coomassie brilliant blue. The purified protein was dialyzed against 20 mM HEPES-NaOH (pH 7.0) and concentrated to 12.6 mg/ml in a buffer containing 20 mM HEPES-NaOH (pH 7.0) and 0.05% Cymal-6.

### Nanodisc preparation.

To assemble CusA into nanodiscs, a mixture containing 20 μM CusA, 45 μM membrane scaffold protein (MSP) (1E3D1), and 930 μM E. coli total extract lipid was incubated for 15 min at room temperature. After the incubation, 1 mg/ml prewashed Bio-Beads (Bio-Rad) were added. The resultant mixture was incubated for 1 h on ice, followed by overnight incubation at 4°C. The protein-nanodisc solution was filtered through 0.22-μm nitrocellulose filter tubes to remove the Bio-Beads. To separate free nanodiscs from CusA-loaded nanodiscs, the filtered protein-nanodisc solution was purified using a Superose 6 column (GE Healthcare) equilibrated with 20 mM HEPES-NaOH (pH 7.0), and fractions corresponding to the size of the trimeric CusA-nanodisc complex were collected for cryo-EM analysis.

### Cryo-EM sample preparation and data collection.

The trimeric CusA nanodisc sample was concentrated to 0.7 mg/ml (2 μM) and incubated with 20 μM Cu(I). The sample was then applied to glow-discharged holey carbon grids (Quantifoil Cu R1.2/1.3, 300 mesh), blotted for 2 s, and then plunge-frozen in liquid ethane using a Vitrobot (Thermo Fisher). The grids were then transferred into cartridges. The images were recorded at 1- to 2.5-μm defocus on a K2 summit direct electron detector (Gatan) with superresolution mode at nominal 81,000 (81K) magnification, corresponding to a sampling interval of 1.08 Å/pixel (superresolution, 0.55 Å/pixel). Each micrograph was exposed for 7.7 s with 5.40 e-/s/physical pixel dose rate (total specimen dose, 50 e^−^/A^2^), and 40 frames were captured per specimen area using Latitude.

### Cryo-EM data processing.

The image stacks in the superresolution model were aligned using cryoSPARC (1). The contrast transfer function (CTF) parameters of the micrographs were determined using Gctf (2). After manual inspection and sorting to discard poor images, ∼1,000 particles were manually picked to generate templates for automatic picking. Initially, 1,769,806 particles were selected after autopicking in cryoSPARC (1). Several iterative rounds of two-dimensional (2D) classifications were performed to remove false picks and classes with unclear features, ice contamination, or carbon. The resulting 549,454 particles were further processed with local motion correction using cryoSPARC and local CTF reestimation by Gctf and used to generate a reference-free *ab initio* three-dimensional (3D) reconstruction. We applied a mask around CusA complex using standard automasking in RELION and generated a mask around the periplasmic cleft of a protomer to use for focused 3D classification. The resulting 3D classes were subjected to 3D reconstruction using an in-house script. For trimeric CusA with one “binding” and two “extrusion” protomers, 75,703 particles were chosen for nonuniform refinement followed by local focused refinement using cryoSPARC resulting in a 2.82-Å global resolution map. For trimeric CusA with two “binding” protomers and one “extrusion” protomer, 43,395 particles were chosen for nonuniform refinement followed by local focused refinement using cryoSPARC resulting in a 3.02-Å global resolution map. For the trimeric pump with three “binding” protomers, 13,304 particles were chosen for nonuniform refinement using cryoSPARC resulting in a 3.40-Å global resolution map based on the gold standard Fourier shell correlation (FSC) (see Table S1 in the supplemental material). For the CusA with three “extrusion” protomers, 21,108 particles were chosen for nonuniform refinement using cryoSPARC resulting in a 3.20-Å global resolution map (Table S1).

### Model building and refinement.

Model building of each trimeric CusA structure was based on the corresponding cryo-EM map. The atomic coordinates of apo-CusA crystal structure (PDB identifier [ID] 3KSS) was fit into the density map using Chimera (3). The subsequent model rebuilding process was performed using Coot (4). Structural refinements were performed using the phenix.real_space_refine program from the PHENIX suite (5, 6). The final atomic model was evaluated using MolProbity (7). The statistics associated with data collection, 3D reconstruction, and model refinement are included in Table S1. Structural refinements were done using the same approach described above (Table S1).

### Molecular modeling and system preparation.

The cryo-EM structures of CusA, which incorporate residues Ile-5 to Trp-1039, were used as the starting point for all simulations. The missing linker between His-425 and Asn-432 in the active-state conformation was modeled into the structures based on the coordinates of the resting state, using a combination of Swiss-Model (23) and PyMOL (Schrodinger LLC, 2015). This made sure that all structures contained an identical amino acid content. The linker between Ile-505 and Asn-515 was not modeled into any of the structures. This yielded a total of 6 models for simulation (Table S2). The simulation systems were initially configured using coarse-grained (CG) molecular dynamics (MD) simulations. The CG simulations were used for the assembly and equilibration of the phospholipid bilayer around the protein. Initially, all structures were converted to CG representation and embedded in a bilayer containing 80% palmitoyl-oleoyl-phosphatidylethanolamine (POPE) and 20% palmitoyl-oleoyl-phosphatidylglycerol (POPG) and solvated in water and 0.15 M NaCl using *insane.py* (24, 25). All simulations were carried out with the MARTINI3 biomolecular force field (26). The tertiary and quaternary structures of the protein were maintained through the application of an elastic network with a force constant of 1,000 kJ mol^−1 ^nm^−2^ between CG backbone particles within 0.5 to 0.9 nm. Systems were energy minimized using the steepest descents algorithm and equilibrated for 1 μs. A temperature of 323 K was maintained throughout the simulation. For all simulations, the V-rescale temperature coupling was used (27), and a pressure of 1 atm was maintained using semi-isotropic Parrinello-Rahman pressure coupling (28). All simulations were carried out using GROMACS-2020 (29).

### All-atom MD simulations.

The final time points of the simulated CG systems were converted to all-atom using CG2AT (https://github.com/owenvickery/cg2at/). For conversion, the original protein model was aligned onto the coordinates of the CG simulation. Simulations were performed with and without the copper(I) ion in their binding site (Table S2) (30). All simulations were carried out using the CHARMM36m biomolecular force field (31). Simulations were energy minimized using the steepest descents algorithm, performed at a temperature of 310 K and equilibrated for 10 ns where the Cα atoms were restrained at 1,000 kJ mol^−1 ^nm^−2^. The production runs of each simulation are listed in Table S2 and were carried out with three repeats. In all simulations, distances were calculated using GROMACS. pK_a_ values were calculated using PROPKATRAJ (32), which uses MDAnalysis to perform the PROPKA3 calculations over the simulation (33). Water wire analysis was carried out using MDAnalysis, based on H-bond analysis (34).

### Accession number(s).

Atomic coordinates and density maps have been deposited with accession numbers or codes 7KF5 (PDB) and EMD-22843 (EMDB) for EEE, 7KF7 (PDB) and EMD-22845 (EMDB) for EEB, 7KF8 (PDB) and EMD-22846 (EMDB) for EBB, and 7KF6 (PDB) and EMD-22844 (EMDB) for BBB.
