# Supplementary figures and images for: Cryo-EM Structures of CusA Reveal a Mechanism of Metal-Ion Export
Source: mBio. 2021 Apr 5;12(2):e00452-21. doi: 10.1128/mBio.00452-21 (PMC8092243; doi:10.1128/mBio.00452-21)

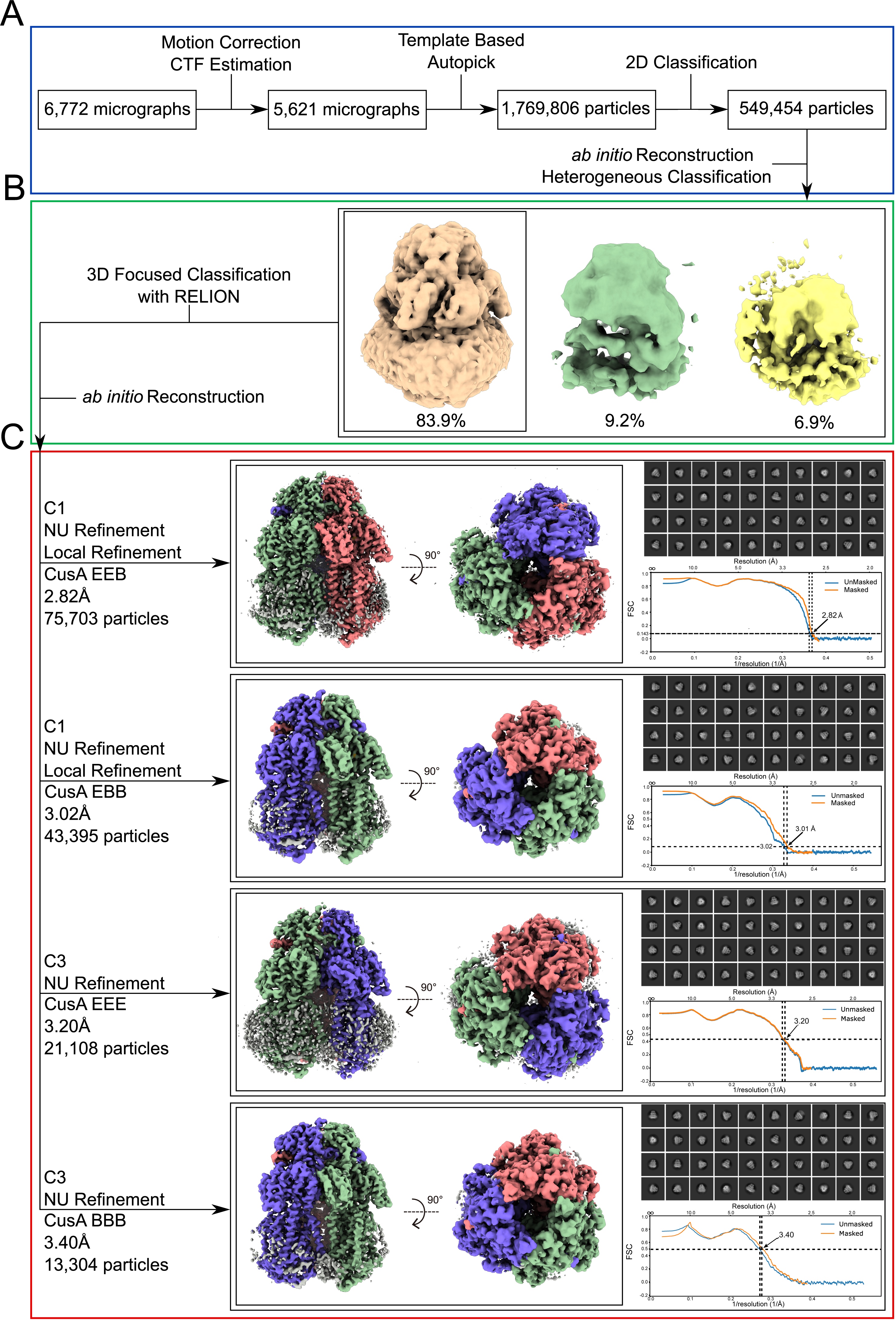

Supplement: FIG S1 [file mBio.00452-21-sf001.jpg]

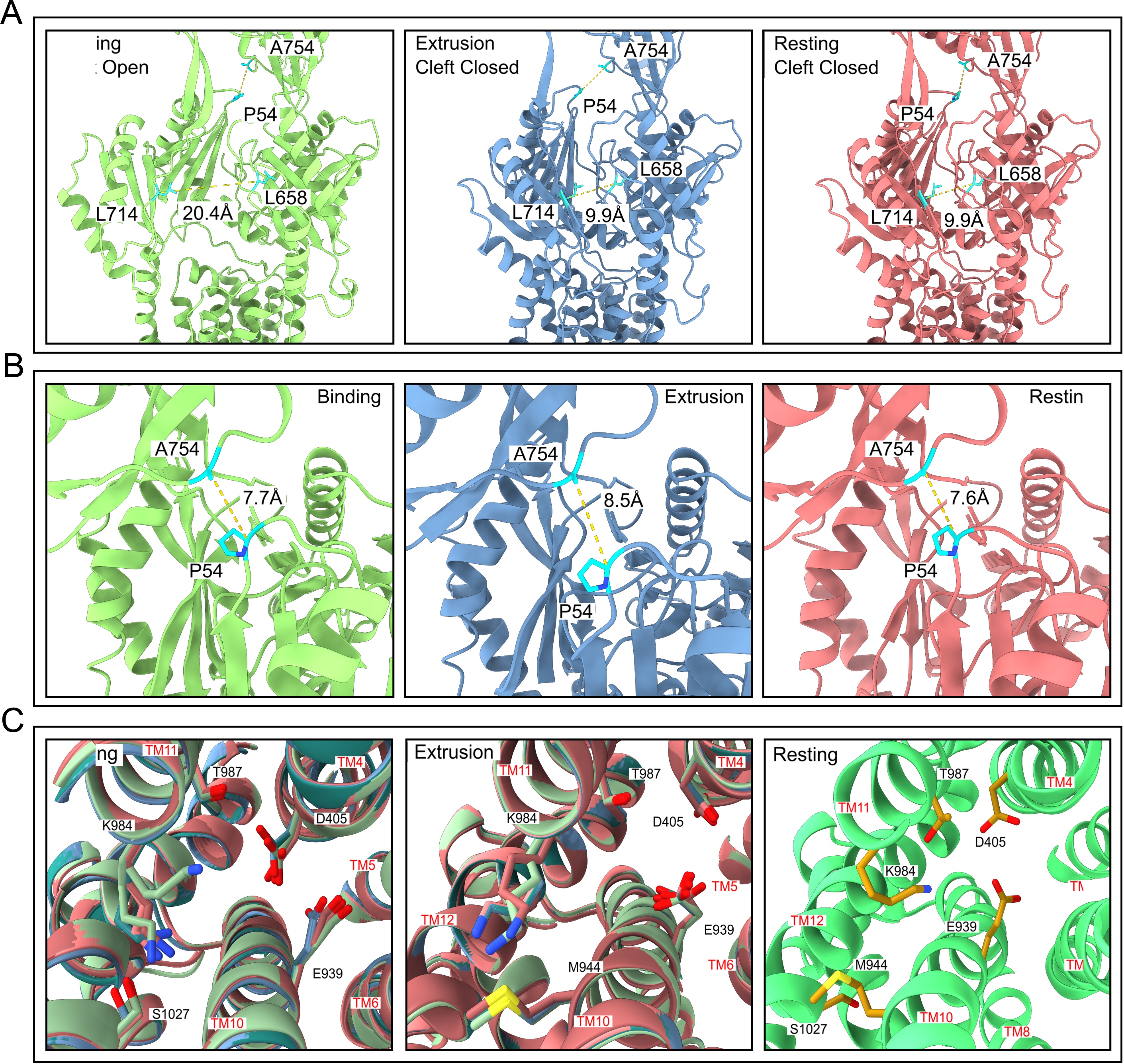

Supplement: FIG S2 [file mBio.00452-21-sf002.jpg]

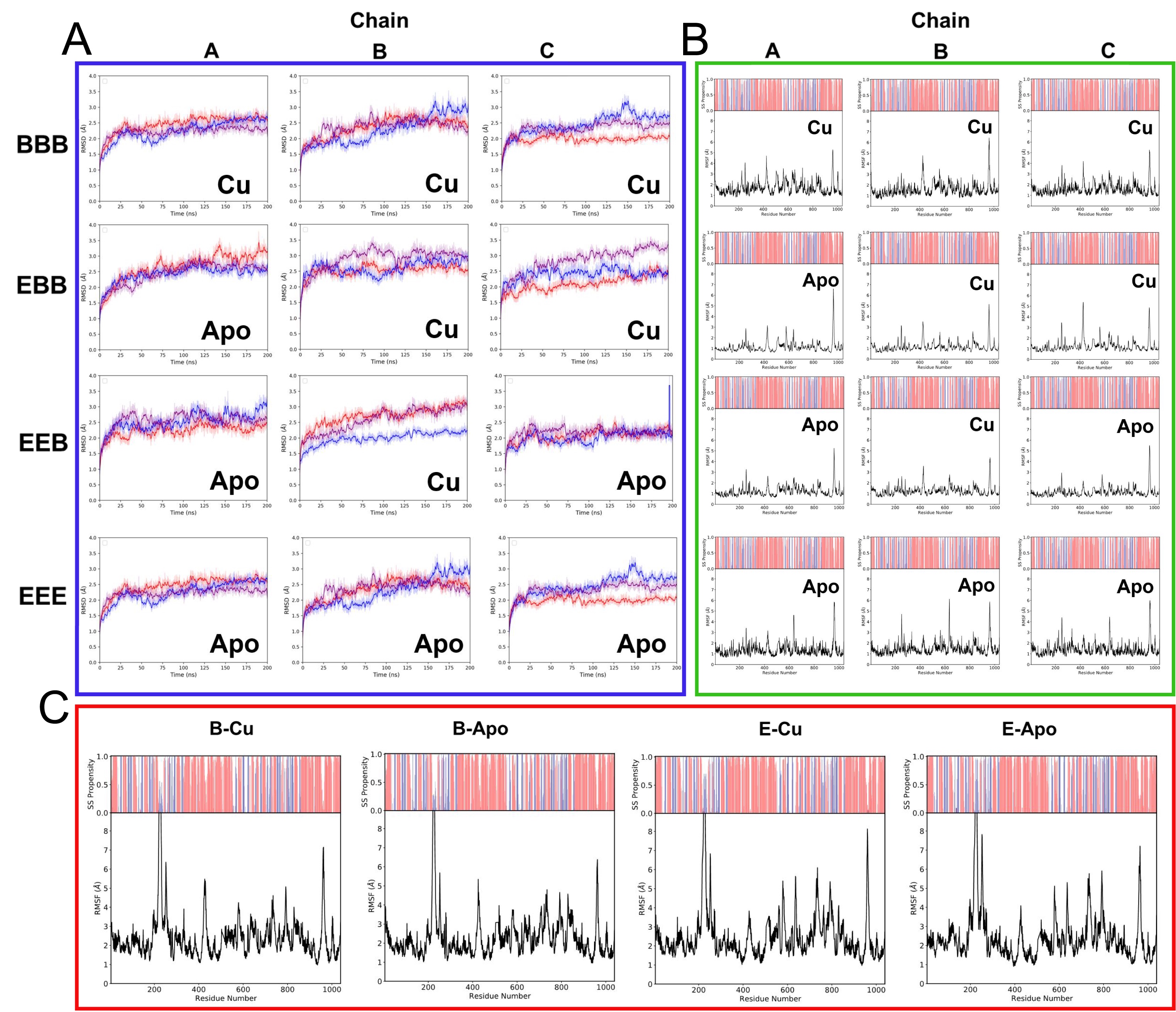

Supplement: FIG S3 [file mBio.00452-21-sf003.jpg]

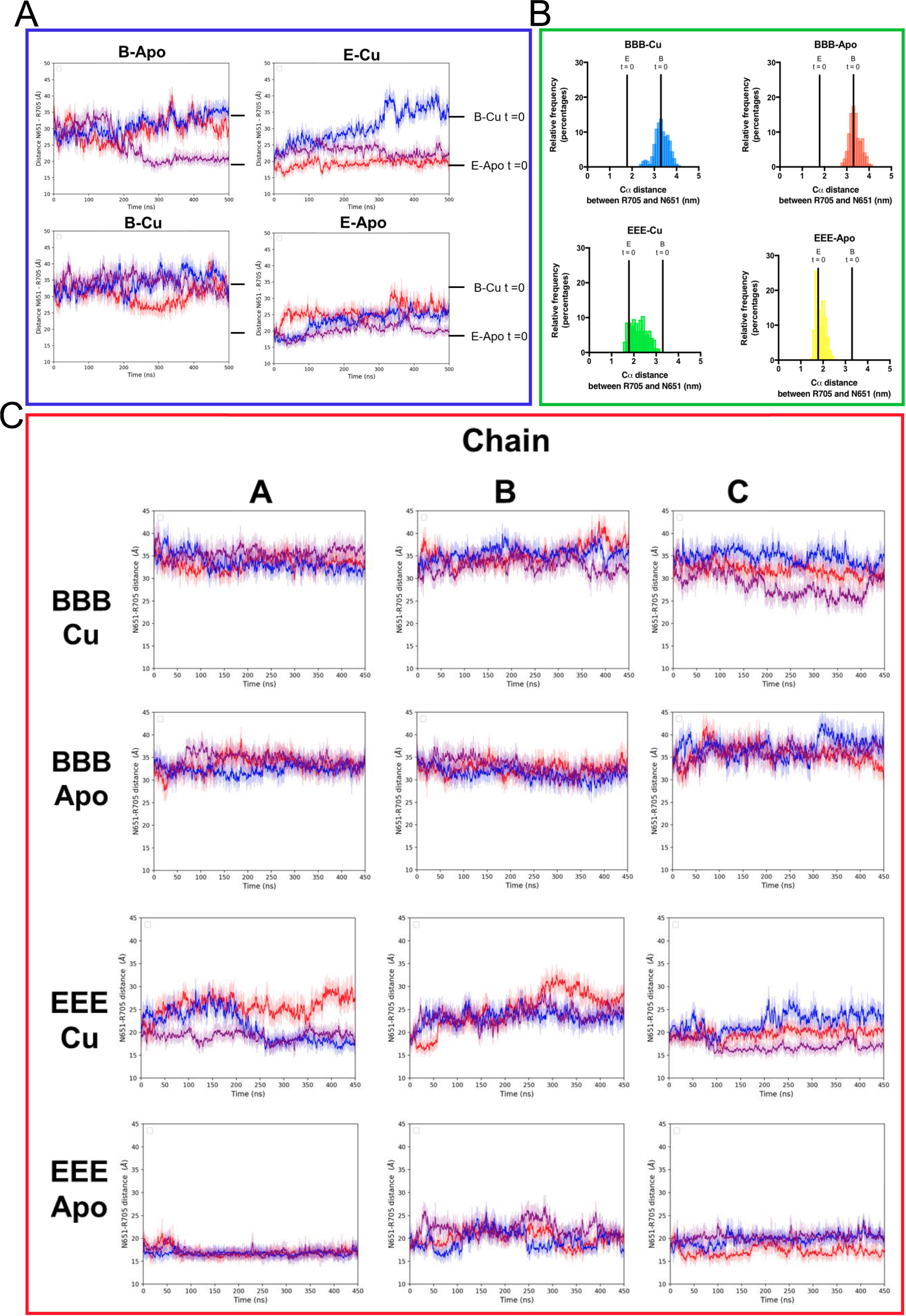

Supplement: FIG S4 [file mBio.00452-21-sf004.jpg]
