# Supplementary material for: Cryo-EM Structures of CusA Reveal a Mechanism of Metal-Ion Export
Source: mBio. 2021 Apr 5;12(2):e00452-21. doi: 10.1128/mBio.00452-21 (PMC8092243; doi:10.1128/mBio.00452-21)
Supplement: TABLE S1 [file mBio.00452-21-st001.pdf]

Table S1. Cryo-EM data collection, processing, and refinement statistics.

PDB ID	7KF5	7KF7	7KF8	7KF6
**Structure**	**EEE (3 Closed)**	**EEB (1 Open 2 Closed)**	**EBB (2 Open 1 Closed)**	**BBB (3 Open)**
Data collection and processing				
Magnification (X)	81,000	81,000	81,000	81,000
Voltage (kV)	300	300	300	300
Electron microscope type	Krios-GIF-K2	Krios-GIF-K3	Krios-GIF-K3	Krios-GIF-K3
Defocus range (μm)	−1.0 to −2.5	−1.0 to −2.5	−1.0 to −2.5	−1.0 to −2.5
Total exposure time (s)	3.3	3.3	3.3	3.3
Energy filter width (eV)	20	20	20	20
Pixel size (Å)	1.08	1.08	1.08	1.08
Total dose (e^-/Å^2)	50	50	50	50
No. of frames	40	40	40	40
Does rate (e^-/Å^2/physical pixel)	7.7	7.7	7.7	7.7
No. of initial micrographs	5,621	5,621	5,621	5,621
No. of initial particle images	549,454	549,454	549,454	549,454
No. of final particle images	21,108	75,703	43,395	13,304
Symmetry	C3	C1	C1	C3
Resolution (Å)	3.20	2.82	3.02	3.40
FSC threshold	0.143	0.143	0.143	0.143
Map resolution range (Å)	2.75 to 8.73	2.38 to 7.36	2.65 to 7.98	2.88 to 9.06
Refinement				
Model resolution cutoff (Å)	3.20	2.82	3.02	3.40
Model composition				
No. of protein residues	3,122	3,122	3,122	3,122
No. of ligands	0	1	2	3
RMSD[a]				
Bond lengths (Å)	0.005	0.005	0.005	0.005
Bond angles (°)	0.790	0.644	0.420	0.420
Validation				
MolProbity score	1.65	1.91	1.65	1.91
Clash score	9.12	6.73	8.34	7.21
Poor rotamers (%)	0	0	0	0
Ramachandran plot (%)				
Favored	96.77	97.40	96.83	97.25
Allowed	3.23	2.60	3.17	2.75
Disallowed	0	0	0	0
CC[b] mask	0.78	0.83	0.80	0.77
CC box	0.74	0.73	0.74	0.73
CC vol	0.76	0.83	0.78	0.76

[a]Root mean square deviation.
[b]Correlation coefficient.
