# Supplementary material for: Cryo-EM Structures of CusA Reveal a Mechanism of Metal-Ion Export
Source: mBio. 2021 Apr 5;12(2):e00452-21. doi: 10.1128/mBio.00452-21 (PMC8092243; doi:10.1128/mBio.00452-21)
Supplement: TABLE S2 [file mBio.00452-21-st002.pdf]

Table S2. Simulations conditions and set-up.

Simulation No.	Starting Structural Models (Abbreviation)	Number of bound Cu(I)	Simulation length (ns)
1	Extrusion state monomer (E)	0	500 x 3
2	Extrusion state monomer (E)	1	500 x 3
3	Bound state monomer (B)	0	500 x 3
4	Bound state monomer (B)	1	500 x 3
5	Extrusion state trimer (EEE)	3	450 x 3
6	Extrusion state trimer (EEE)	0	450 x 3
7	2:1 extrusion/bound state trimer (EEB)	2	200 x 3
8	1:2 extrusion/bound state trimer (EBB)	1	200 x 3
9	Bound state trimer (BBB)	0	450 x 3
10	Bound state trimer (BBB)	3	450 x 3
		**Total**	**12.6 µs**
